# Fecal parasite risk in the endangered proboscis monkey is higher in an anthropogenically managed forest environment compared to a riparian rain forest in Sabah, Borneo

**DOI:** 10.1371/journal.pone.0195584

**Published:** 2018-04-09

**Authors:** Annette Klaus, Christina Strube, Kathrin Monika Röper, Ute Radespiel, Frank Schaarschmidt, Senthilvel Nathan, Benoit Goossens, Elke Zimmermann

**Affiliations:** 1 Institute of Zoology, University of Veterinary Medicine Hannover, Hannover, Lower Saxony, Germany; 2 Institute for Parasitology, Centre for Infection Medicine, University of Veterinary Medicine Hannover, Hannover, Lower Saxony, Germany; 3 Institute for Biostatistics, Leibniz University, Hannover, Lower Saxony, Germany; 4 Sabah Wildlife Department, Kota Kinabalu, Sabah, Malaysia; 5 Organisms and Environment Division, School of Biosciences, Cardiff University, Cardiff, Wales, United Kingdom; 6 Danau Girang Field Centre, c/o Sabah Wildlife Department, Kota Kinabalu, Sabah, Malaysia; 7 Sustainable Places Research Institute, Cardiff University, Cardiff, Wales, United Kingdom; Universitat Autonoma de Barcelona, SPAIN

## Abstract

Understanding determinants shaping infection risk of endangered wildlife is a major topic in conservation medicine. The proboscis monkey, *Nasalis larvatus*, an endemic primate flagship species for conservation in Borneo, is endangered through habitat loss, but can still be found in riparian lowland and mangrove forests, and in some protected areas. To assess socioecological and anthropogenic influence on intestinal helminth infections in *N*. *larvatus*, 724 fecal samples of harem and bachelor groups, varying in size and the number of juveniles, were collected between June and October 2012 from two study sites in Malaysian Borneo: 634 samples were obtained from groups inhabiting the Lower Kinabatangan Wildlife Sanctuary (LKWS), 90 samples were collected from groups of the Labuk Bay Proboscis Monkey Sanctuary (LBPMS), where monkeys are fed on stationary feeding platforms. Parasite risk was quantified by intestinal helminth prevalence, host parasite species richness (PSR), and eggs per gram feces (epg). Generalized linear mixed effect models were applied to explore whether study site, group type, group size, the number of juveniles per group, and sampling month predict parasite risk. At the LBPMS, prevalence and epg of *Trichuris* spp., strongylids, and *Strongyloides* spp. but not *Ascaris* spp., as well as host PSR were significantly elevated. Only for *Strongyloides* spp., prevalence showed significant changes between months; at both sites, the beginning rainy season with increased precipitation was linked to higher prevalence, suggesting the external life cycle of *Strongyloides* spp. to benefit from humidity. Higher prevalence, epgs, and PSR within the LBPMS suggest that anthropogenic factors shape host infection risk more than socioecological factors, most likely via higher re-infection rates and chronic stress. Noninvasive measurement of fecal parasite stages is an important tool for assessing transmission dynamics and infection risks for endangered tropical wildlife. Findings will contribute to healthcare management in nature and in anthropogenically managed environments.

## Introduction

Parasite infection patterns are shaped by multifaceted variables. Understanding the drivers of parasitism is a main goal of conservation medicine [1] and crucial to assess the impact of parasites and diseases on endangered species [2, 3]. Especially nonhuman primate populations are increasingly threatened by massive anthropogenic changes of natural habitats. These changes have recently been shown to also induce alterations of parasite risks on nonhuman primate reproduction and survival [4, 5, 6].

Anthropogenic influence was described to promote helminth infection measures indirectly via different modes of intervention, such as provisioning of food resources leading to increased host contact and parasite transmission rates [7, 8]. In natural forests affected by fragmentation, host population density was documented to correlate positively with parasite richness and prevalence [9]. In different forest types, it was shown that the risk of infection may rise with the presence of more social group partners within a group [10], but that larger groups may also disrupt direct transmission dynamics through fission as a counter-strategy [11, 12]. Additionally, host age and developing immunity [13] as well as co-infection status [14] may affect parasite risk. Furthermore, hormonally mediated sex differences may lead to divergent infection patterns in different types of social groups like harem (one male-multi female) and bachelor (all male) units, as immunosuppressive steroid hormone levels were stated to cause a male bias in parasitism [15]. Moreover, it was shown that climatic changes and seasonal challenges in moist and dry tropical forests shape parasite risk; increased precipitation may promote survival of specific parasite intermediates [16] whereas drier habitat conditions may influence host susceptibility towards parasite infections [17].

**Fig 1:**
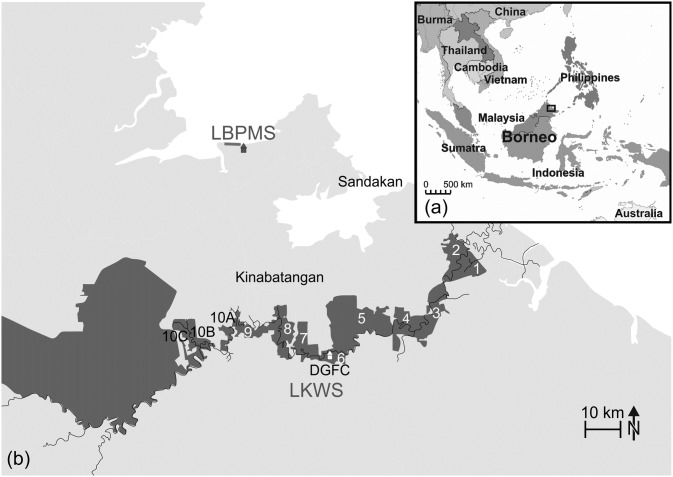
Sample collection sites in Sabah, Malaysian Borneo, Southeast Asia. (a) Map with Borneo, Southeast Asia, and the position of sampling sites framed. (b) Enlarged map with Sabah, Malaysian Borneo, and the position of the two sampling sites: the LKWS with Lots 1–10 (dark grey) along the Kinabatangan River (black line) with DGFC (white house) in Lot 6, and the LBPMS near the coastline (grey house). LKWS = Lower Kinabatangan Wildlife Sanctuary, LBPMS = Labuk Bay Proboscis Monkey Sanctuary, DGFC = Danau Girang Field Centre.

Borneo is known to harbor a vast number of endemic plant and animal species threatened by deforestation [18]. The proboscis monkey (*Nasalis larvatus*) is a primate flagship species for conservation in Sabah [19, 20]. Linked to lowland riverine and coastal mangrove forests, proboscis monkeys inhabit a habitat type most affected by logging and agricultural land use [21]. Social groups consist of up to 30 individuals and are organized as either harems (one male-multi female-groups with their offspring) or bachelor (all male) units [22]. Since the 1990s, proboscis monkeys became a major focus for ecotourism [23]. The Lower Kinabatangan Wildlife Sanctuary (LKWS) is a protected area open for tourism and some of the well-known wildlife tourism projects include the Labuk Bay Proboscis Monkey Sanctuary (LBPMS) [24]. The LKWS is situated along the floodplain of the Kinabatangan River in Sabah, Malaysian Borneo, and consists of 10 blocks of remaining forest, so called ‘Lots’, along the river (Fig 1). Most of the area surrounding the LKWS was converted to oil palm plantations during the last decades. Local proboscis monkey groups (Table 1) can range between adjacent Lots or cross the Kinabatangan [25]. The LBPMS comprises private land near Samawang Village at Labuk Bay, Sandakan, in the northeast of Malaysian Sabah. It consists of a stretch of mangrove forest and coastal swamps along the coastline [26] and is further surrounded by agriculture (Fig 1). The anthropogenically managed sanctuary shelters a population of proboscis monkeys organized in six harems and three bachelor groups (personal communication of rangers in 2012) (Table 1).

**Table 1:** Local proboscis monkey density across two sample collection sites in Sabah, Malaysian Borneo.

Sampling site	Size of location (ha)	No. of proboscis monkeys	Proboscis monkey density^a^
LKWS	27,000 [27]	1,400–3,400 [28]	0.05–0.12
LBPMS	160 [26]	160–190^b^	1–1.2

Ha = hectare.

No. of proboscis monkeys = Estimated number of proboscis monkey individuals per sampling site.

^a^ estimated proboscis monkey density given as individuals per ha.

^b^ according to personal communication of rangers in 2012.

LKWS = Lower Kinabatangan Wildlife Sanctuary, Lots 1–10.

LBPMS = Labuk Bay Proboscis Monkey Sanctuary.

Noninvasive rapid assessment tools for estimating parasite risks are of imminent importance for proboscis monkeys, since parasites may be a risk for survival of this endangered monkey species. Here, we explore whether the noninvasive collection of fecal samples can be used to assess the relative impact of anthropogenic (site of origin) and ecological parameters (season = time of sampling/month of the year; group size; group type = harem or bachelor, and group demography = proportion of juveniles per group) on parasite risk in the LKWS and the LBPMS. Host parasite risk was quantified by parasite prevalence, parasite species richness, and egg shedding intensity. The following hypotheses will be explored: (1) study site has a significant effect on parasite risk: parasite prevalence, species richness, and egg shedding intensity are higher in the forest area with feeding platforms and higher host density (LBPMS); (2) group size, group type or demography will shape parasite risk: larger groups or bachelor groups have a generally higher parasite prevalence, species richness, and epgs, whereas groups with more juveniles show a higher prevalence of typical juvenile parasitoses; (3) season will affect parasite risk specifically according to parasite transmission cycles: prevalence of parasite species which benefit from humidity increases with rainfall in particular months of the year.

## Materials and methods

### Sampling sites

**Fig 2:**
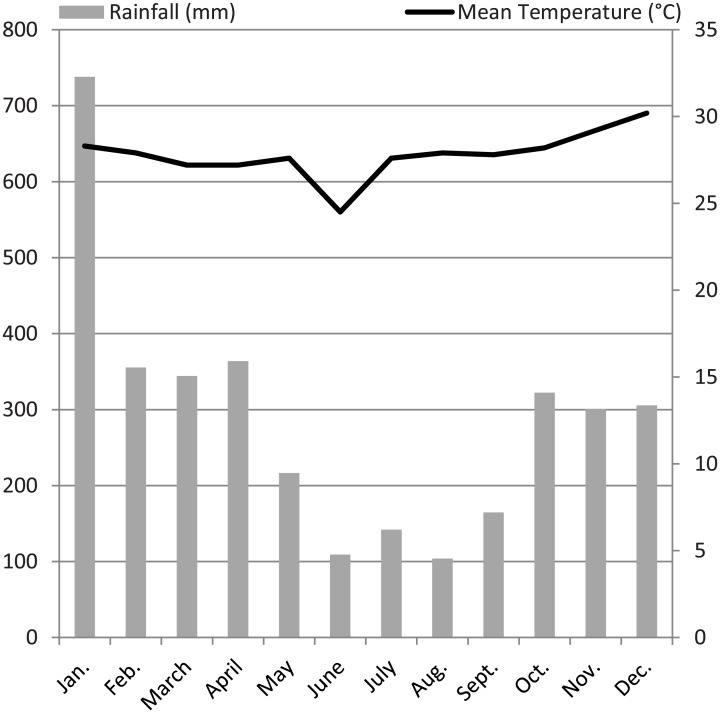
Climate diagram of 2012 based on data from the Danau Girang Field Centre in Lot 6 of the Lower Kinabatangan Wildlife Sanctuary (LKWS) in Sabah.

In Sabah, Malaysian Borneo, mean temperature variation over the year varies monthly between 21° and 34°C [29]. The annual precipitation inland is 2,800 mm on average [30] and is influenced by two yearly raining seasons; the stronger raining season usually lasts from October to March and the weaker raining season from April to September (Fig 2) [31].

#### Lower Kinabatangan Wildlife Sanctuary (LKWS)

Sampling was conducted in Lot 6 of the government-controlled conservation area of the LKWS (5° 24.231’-5° 24.985’N; 117° 59.682’-118° 04.084’E) and always started at the Danau Girang Field Centre (DGFC) (Fig 1). Using ACME Planimeter measuring the area in Google Maps, the estimated size of Lot 6 was determined as 1,870 ha (http://www.acme.com/planimeter, retrieved 3^rd^ November 2016). According to repeated surveys close to DGFC and a nearby tributary, about 15 groups of proboscis monkeys were inhabiting Lot 6 in 2010 [28]. For the present study, boat surveys for the localization of groups and subsequent sample collection were conducted 4.7 km downstream (3.0 km linear distance) and 6.1 km upstream (3.7 km linear distance) in 2012 along the south river bank starting from DGFC.

#### Labuk Bay Proboscis Monkey Sanctuary (LBPMS)

Sample collection took place near feeding platforms on the private grounds of the LBPMS close to Samawang Village at Labuk Bay, Sandakan (Fig 1). Here, at two public feeding platforms A (5° 56.130’N; 117° 48.570’E) and B (5° 56.188’N; 117° 47.880’E), free-ranging groups are offered supplementary food, namely man-made cake and cucumber [32] by staff of the LBPMS twice a day. Proboscis monkey groups visited these artificial feeding platforms regularly, most often shortly before being fed.

### Sample collection

During the end of the dry and beginning of the rainy season from June to September 2012, a total of 634 fecal samples (6-56/group/day) were obtained in the LKWS, originating from 16 sampling events from harem groups and four sampling events from bachelor groups (Table 2). The weather conditions, in particular storms and rainfall, strongly influenced the feasibility of boat excursions, localization of groups, and successful sample collection and are responsible for the varying number of samples per month. Before sunset (17:00 h– 19:00 h local time), groups near the river bank were localized because proboscis monkeys commonly return to riverside trees for sleeping [33]. Until group members fell asleep, data on group type, group size, the number of juvenile group members, and GPS coordinates were documented. On the next day before sunrise (5:00 h– 8:00 h local time), the same group was approached again to collect as many fresh fecal samples per group per day as possible. Immediately after the animals woke up and foraged deeper into the forest, collection of samples took place. Samples obtained from different days were treated as disparate sampling units. Home ranges of proboscis monkey groups can extensively overlap [34]. Therefore, individual identification of groups was uncertain. Nevertheless, based on our observations of group sizes and group composition, the distribution of groups along the Kinabatangan River, and published estimates of home ranges [35], we assume that we sampled at least nine different harem groups and three different bachelor groups. Therefore, we expect that most of the daily group samples stemmed from different social groups and only a minority of group samples would constitute resampling events.

**Table 2:** Distribution of the number of monthly fecal samples collected from proboscis monkeys at two sampling sites in Malaysian Borneo in 2012.

Sampling site (no. of total fecal samples)	Month	Number of samples/month	Number of sampling events from harem groups/month	with a no. of juveniles <0.3	with a no. of juveniles >0.3	with a no. of juveniles NA	Group size range of sampled harem groups	Number of sampling events from bachelor groups^a^/month	Group size range of sampled bachelor groups
LKWS (634)	June	357	9	1	7	1	5–22	2	4–9
	July	183	3	1	2	0	4–11	2	8–8
	August	61	2	2	0	0	10–14	0	-
	September	33	2	0	2	0	13–17	0	-
LBPMS (90)	September	38	3	1	2	0	21–36	2	13–19
	October	52	4	2	1	1	21–36	2	13–19

No. of juveniles = Number of juveniles (given as the proportion of juvenile group members) per group.

NA = information not available.

Group size range = minimum and maximum number of individuals per group.

^a^ bachelor groups do not contain juvenile group members.

LKWS = Lower Kinabatangan Wildlife Sanctuary.

LBPMS = Labuk Bay Proboscis Monkey Sanctuary.

Between 28^th^ September and 18^th^ October 2012, 12 days of feces collection were conducted at the LBPMS. In total, 90 fecal samples (1-18/group/day) were collected during seven sampling events from three harem groups and during four sampling events from two bachelor groups (Table 2) which could be individually identified with the help of the local rangers. Fecal samples were taken after five feeding sessions on platform A (9:30 h local time) and six feeding sessions on platform B (16:30 h local time) when the animals did leave the platforms and returned into the mangroves. During each feeding session observations, data were collected on group type, group size, and the number of juvenile group members.

### Treatment of fecal samples and sample analyses

In order to impede a contamination with parasite stages from the ground, the surface of each fecal sample was removed with the help of a spatula. Collected in a 15 mL tube, each fecal sample was weighed and preserved in 10% buffered formalin in a 1:3 ratio (for 1 L: 900 mL distilled water, 100 mL formaldehyde 37–40%, 6.5 g anhydrous disodium hydrogen phosphate Na_2_HPO_4_, 4.0 g sodium dihydrogen phosphate NaH_2_PO_4_). Analysis of fecal samples was performed using a modified sodium nitrate flotation method [36]. Sodium nitrate solution was made of 600 g sodium nitrate (NaNO_3_) dissolved in 1 L distilled water for a specific gravity of 1.3, which was steadily verified with a hydrometer (Hydrometer g/mL Tp. 20°C 55mN/m ST. N° 0310, ALLA France). At first, the preservative formalin was largely discarded from the feces by centrifugation at 700 x *g* for 10 minutes (Heraeus Multifuge 3 S-R, Thermo Fisher Scientific, Waltham, MA, USA). Supernatant was removed and feces were washed through homogenization in distilled water and centrifuged a second time. Subsequently, from each sample a maximum of two gram of feces were mixed with sodium nitrate filling up the sample tube up to 15 mL and centrifuged at 860 x *g* for 10 minutes. With the help of an eyelet, the whole liquid surface was transferred onto a slide and covered with a coverslip. With an Axiophot microscope (Carl Zeiss MicroImaging, Oberkochen, Germany) each sample was instantly examined microscopically at x10 and x40 magnification. To determine the number of eggs per gram feces, all detected parasitic stages were counted. Using an Olympus ColorView **III**u digital camera (OLYMPUS Soft Imaging Solutions, Munster, Germany), detected parasitic stages (eggs only) were photographed and subsequently measured with the software cell^ B (version 3.1, Olympus Soft Imaging Solutions). In order to avoid bias during investigations, analyses of samples were conducted in a randomized order.

### Data analyses

Three parameters of parasite risk were analyzed and tested as dependent variables; (1) parasite prevalence (number of infected individuals as percentage of the whole number of examined animals), (2) host parasite species richness (number of different parasite species morphotypes found in a single host, PSR), and (3) host parasite egg shedding intensity (number of helminth eggs per gram collected feces, epg). Due to the extremely low observed prevalence (0.2%), *Trichuris* morphotype T4 was excluded from statistical analyses. In total, 724 fecal samples were included in the analyses; 634 samples were from the LKWS and 90 samples from the LBPMS.

To investigate variables influencing prevalence of nematode infections and PSR, two-step generalized linear mixed models (GLMMs) were calculated for each helminth group (a) using binomial presence/absence data (for prevalence) and (b) under the assumption of a Poisson distribution (for PSR). To control for the possibility that wild groups were sampled repeatedly, sampling events (= sampled groups of proboscis monkeys) were set as a random factor in each model. Model comparison and selection were conducted via the *anova* function (analysis of variance) using the packages lme4, version 1.1–10 [37] and effects [38] in the R software environment (version 0.97.551, R Core Team). In preliminary analyses, all independent variables were first tested separately. In the next step of analyses, the influence of sample weight and sampling site (LKWS *vs*. LBPMS) on parasite risk were tested. Sample weight might affect detection rates of parasite stages shed by species with low levels of egg shedding, e.g. *Strongyloides* spp. [39]. Furthermore, sample weight could have a diluting effect on high numbers of egg shedding, e.g. of *Trichuris* spp. [40]. If sample weight had a significant influence on the dependent variable, it was retained in the following models; otherwise it was no longer included. If the sampling site had a significant effect, the subsequent models were run separately for each sampling site; otherwise one joint model was calculated. In the next step, full models were built that contained at least four fixed factors that could be of biological relevance: (1) group type (harem/bachelor), (2) group size, (3) proportion of juveniles per group (0; <0.3; >0.3), and (4) time of sampling = month of the year (June; July; August; September; October). In addition, an interaction between group size and the proportion of juveniles was also included for typical juvenile parasitoses, such as *Strongyloides* spp. and *Ascaris* spp. infections [41] in a third step. Likewise, the interaction term was included in statistical models for PSR. If the inclusion of this term improved the model, it was retained; if not, the interaction term was excluded from the model. Whenever the independent variable (4) time of sampling was a significant predictor variable in analyses for samples collected from the LKWS, all months were compared with a post hoc Tukey-test using the package multcomp [42] to identify significant pairwise differences between sampling months.

Epg data were log(1+x)-transformed and analyzed via two-step linear mixed effect models accordingly, with (1) sampling site, (2) group type, (3) group size, (4) proportion of juveniles per group as well as (5) month of the year as fixed effects. Analysis of model residuals and random effect estimates in QQ-plots showed that a normal distribution on the log-scale was tenable only for most abundant taxonomic groups (*Trichuris* spp. and strongyles). For the rarer taxonomic groups residuals were clearly right-skewed after log-transformation (*Strongyloides* spp., *Ascaris* spp., *Enterobius* spp., and *Anatrichosoma* spp.).

### Ethics statement

The authors confirm that they did not interact with or disrupt the proboscis monkeys in any way. Fecal sample collection was performed noninvasively from free-ranging individuals without direct animal contact or disturbing the animals and adhered to the Code of Best Practices for Field Primatology of the International Primatological Society (IPS), the ethical guidelines of the German Primate Society (GfP), and the German Animal Protection Act. Sampling in this study required approval and was authorized by the Sabah Wildlife Department, the Sabah Biodiversity Council, the Danau Girang Field Centre as well as the Labuk Bay Proboscis Monkey Sanctuary and complied with Malaysia’s law on foreign research (Licence N° UPE: 40/200/19/2822).

## Results

### General parasite diversity across both study sites

Overall, 10 helminth egg morphotypes were identified in fecal samples collected from both sites, the LKWS and the LBPMS; namely four different morphotypes of *Trichuris* spp. (T1-T4) as well as eggs from *Anatrichosoma* spp., *Trichostrongylus* spp. [43], *Oesophagostomum*/*Ternidens* spp. [41, 44], an unknown strongylid, *Strongyloides* spp., and *Ascaris lumbricoides* [45]. In addition, *Enterobius* spp. eggs were detected in samples from the LKWS [14].

### Parasite prevalence

**Fig 3:**
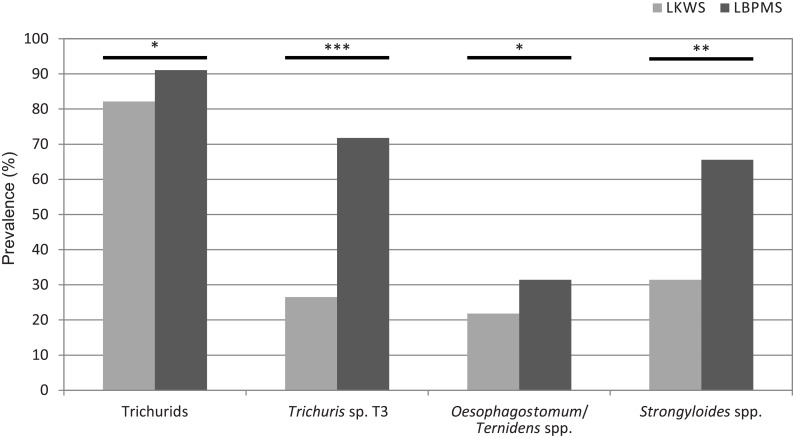
Prevalence of intestinal helminth parasites found in proboscis monkey feces at the two sampling sites. LKWS = Lower Kinabatangan Wildlife Sanctuary, LBPMS = Labuk Bay Proboscis Monkey Sanctuary. * p≤0.05; ** p≤0.001; *** p≤0.0001.

Overall parasite prevalence was linked to parasite taxa and ranged between 1.46 and 91.11% across study sites (Table 3). For trichurids, in particular *Trichuris* morphotype T3 as well as for *Oesophagostomum*/*Ternidens* spp. and *Strongyloides* spp., but not for the other helminth taxa, prevalence was significantly higher at the LBPMS than at the LKWS (Fig 3, S1 Table). Sample weight had a significant effect on the prevalence of trichurids, namely *Trichuris* spp. T1-T3, the unknown strongylid, and *Strongyloides* spp. and was therefore included as a fixed effect in respective statistical models (S1 Table). No significant effect of the variables group type, group size, and the proportion of juvenile group members on helminth prevalence was observed.

**Table 3:** Prevalence of different helminth egg morphotypes in fecal samples from proboscis monkeys at two sampling sites in Malaysian Borneo.

	LKWS (n = 634) prevalence (%)					LBPMS (n = 90) prevalence (%)		
	June (n = 357)	July (n = 183)	August (n = 61)	September (n = 33)	Overall (n = 634)	September (n = 38)	October (n = 52)	Overall (n = 90)
Trichurids	83.47	81.42	72.13	90.90	**82.17**	86.84	94.23	**91.11**
*Trichuris* sp. T1^a^	13.52	7.42	6.55	8.33	**10.89**	15.38	13.33	**14.08**
*Trichuris* sp. T2^a^	81.97	78.28	67.21	79.16	**79.34**	73.07	84.44	**80.28**
*Trichuris* sp. T3^a^	25.07	28.57	24.59	37.50	**26.50**	73.07	71.11	**71.83**
*Anatrichosoma* spp.^a^	1.12	2.28	0.00	4.16	**1.46**	0.00	4.44	**2.81**
Strongylida	53.78	62.29	57.37	75.75	**57.72**	36.84	48.07	**43.33**
*Trichostrongylus* spp.^b^	44.61	51.74	44.26	56.00	**47.12**	32.43	38.77	**36.04**
*Oesophagostomum*/*Ternidens* spp.^b^	18.26	26.16	24.59	32.00	**21.79**	27.02	34.69	**31.39**
Unknown strongylid^b^	2.69	2.90	0.00	12.00	**3.21**	5.40	4.08	**4.65**
*Strongyloides* spp.	25.77	32.24	49.18	54.54	**31.38**	52.63	75.00	**65.55**
*Ascaris lumbricoides*	9.80	8.74	1.63	0.00	**8.20**	5.26	0.00	**2.22**
*Enterobius* spp.	7.28	2.73	8.19	0.00	**5.67**	0.00	0.00	**0.00**

LKWS = Lower Kinabatangan Wildlife Sanctuary, LBPMS = Labuk Bay Proboscis Monkey Sanctuary.

^a^ considered sample size of trichurid species: LKWS = 615, June = 355, July = 175, September = 24; LBPMS = 71, September = 26, October = 45.

^b^ considered sample size of strongylid species: LKWS = 592, June = 334, July = 172, September = 25; LBPMS = 86, September = 37, October = 49.

The time of sampling (month of the year) had no significant effect on the prevalence of helminth species at the two sampling sites, except for *Strongyloides* spp., in which prevalence significantly increased from June on towards September/October at both sampling sites (S1 Table).

### Parasite species richness (PSR)

**Fig 4:**
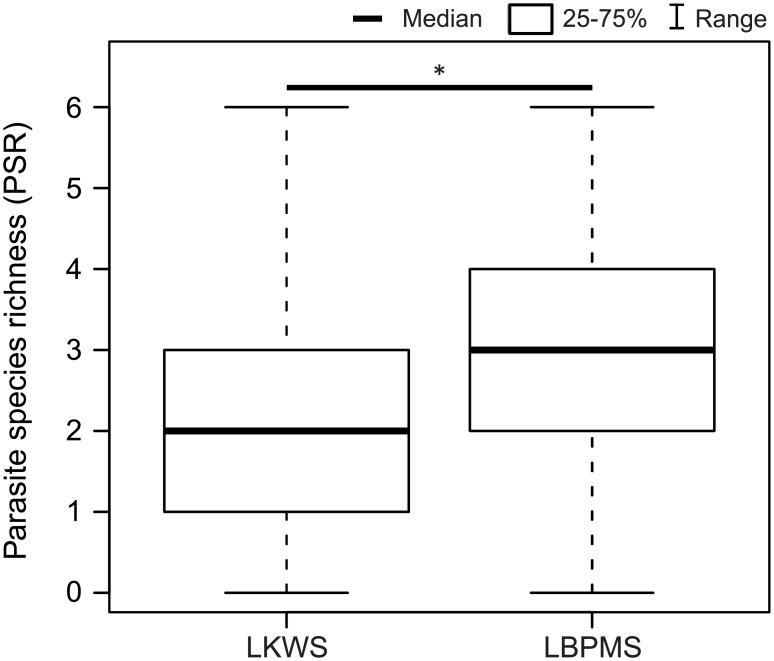
Parasite species richness of proboscis monkeys at two sampling sites in Sabah. LKWS = Lower Kinabatangan Wildlife Sanctuary, LBPMS = Labuk Bay Proboscis Monkey Sanctuary. Band in boxes is the median, boxes define the 25th and 75th percentiles, whiskers extend to maximum +/- 1.5 times the interquartile range (IQR = middle 50% of the records). * p≤0.05; ** p≤0.001; *** p≤0.0001.

Parasite species richness was significantly higher in the LBPMS than in the LKWS (Fig 4). Among fecal samples analyzed for different egg morphotypes of helminths (596), 131 samples contained eggs of one morphotype (22.0%), 172 samples contained eggs of two morphotypes (28.9%), 158 samples had eggs of three morphotypes (26.5%), 91 showed eggs of four morphotypes (15.3%), 33 samples contained eggs of five morphotypes (5.5%), and eleven samples had eggs of six morphotypes (1.8%). Overall mean PSR was 2.6 (± 1.2 SD). For the variables group type, group size, and the proportion of juvenile group members no significant effect on PSR was identified (S2 Table).

### Egg shedding intensity

**Fig 5:**
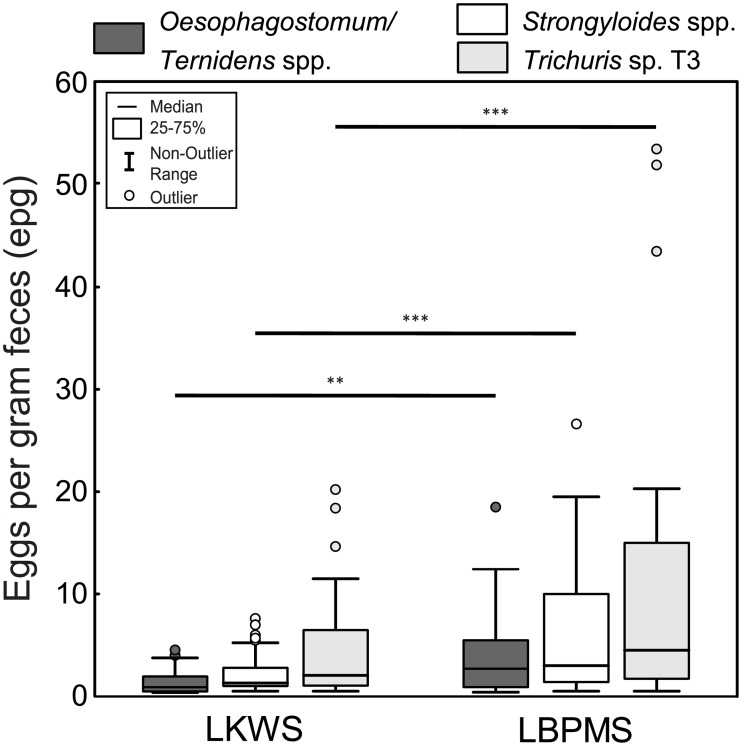
Eggs per gram feces (epg) of positive *Oesophagostomum/Ternidens* spp., *Strongyloides* spp., and *Trichuris* sp. T3 fecal samples from proboscis monkeys at two sampling sites in Sabah. LKWS = Lower Kinabatangan Wildlife Sanctuary, LBPMS = Labuk Bay Proboscis Monkey Sanctuary. Band in boxes is the median, boxes define the 25th and 75th percentiles, whiskers extend to maximum +/- 1.5 times the interquartile range (IQR = middle 50% of the records). * p≤0.05; ** p≤0.001; *** p≤0.0001.

Egg shedding intensity (eggs per gram feces epg) was linked to helminth taxa and sampling site had a significant effect on the epg values of *Trichuris* sp. T3, *Oesophagostomum/Ternidens* spp., and *Strongyloides* spp. but not on the other helminth taxa (Fig 5, S3 Table). In all cases, egg shedding intensity was significantly higher in the LBPMS than in the LKWS. Neither the variables group type, group size, the proportion of juvenile group members nor the time of sampling significantly influenced epg values.

## Discussion

Our study shows for the first time that fecal collection provides an important noninvasive tool to assess parasite risk of endangered wildlife in different tropical forest settings of Borneo using the proboscis monkey.

As expected, study site was the strongest predictor variable of the intestinal parasite risk in this large bodied primate. This finding suggests that an anthropogenically managed forest area may facilitate intestinal parasite transmission between hosts. Several factors may explain this difference between study sites. Studies on various nonhuman primate species showed that host density is a key determinant for parasite infections [5, 46] and that human provisioning of wildlife with food further affects parasite spread through induced host aggregation and proximity [47]. Based on published estimates for proboscis monkeys along the LKWS [28], and own assessments at the LBPMS, the population density with about 1–1.2 individuals/ha is more than 10 times higher than in Lot 6 of the LKWS with 0.09 individuals/ha. An increased overall host density may cause an accumulation of infective soil-transmitted parasite stages in the environment, which favors the outbreak of significantly more and stronger (re-)infections [48, 49]. The concentrated feeding regime in the LBPMS which is based on a small number of feeding platforms is furthermore increasing encounter rates and the potential exchange of parasite stages via the joint use of the same substrate. The enhanced prevalence of trichurid morphotypes, *Oesophagostomum*/*Ternidens* spp., and *Strongyloides* spp. at the LBPMS goes hand in hand with significantly increased numbers of eggs per gram feces (epg) for *Trichuris* sp. T3, *Oesophagostomum*/*Ternidens* spp., and *Strongyloides* spp. as well as a significantly higher individual parasite species richness (PSR). An enhanced host density can also be associated with a higher competition for food, sleeping sites, and mating partners as well as frequent contact to visitors and staff, and thus may cause stress, immunosuppression [50] and a higher risk for worm infection. Our findings coincide with those on Thai long-tailed macaques (*Macaca fascicularis*) which showed increased *Strongyloides* spp. prevalence and epgs in an anthropogenically modified environment, correlated with higher portions of human-provided food and the time spend foraging on the ground [8]. Likewise, in other wildlife like wild boar (*Sus scrofa*), anthropogenic management with supplemental feeding was proven to drive the probability and intensity of gastrointestinal parasite infections as well as parasite species richness [51]. Future studies should additionally focus on dietary differences between the proboscis monkey populations. It is well known for mammals that a varying nutritional uptake affects the structure of the gut’s microbiome [52], which itself was recently shown to interact with the intestinal parasite fauna [53]. Ingredients of man-made cake which is supplementary fed at the LBPMS, like carbohydrates from wheat and rice flour [32], may thus be potentially related to the observed parasite infection patterns as well.

In the literature, ecological factors like social group size [54], group demography, and seasonal climatic variations [55] are discussed to predict parasite risk. Here, we considered the potential impact of several ecological parameters on parasite risk for proboscis monkeys at the LKWS and the LBPMS with the help of generalized linear mixed effect models. No effect of the variable group size was observed in any of the parasite types present in this study. Results could potentially be explained by social dynamics, as fission-fusion patterns of proboscis monkey groups could lead to temporally changing group densities or sizes [56], masking potential effects of individual group sizes on infection risk. However, our findings correspond to results on red colobus (*Procolobus rufomitratus*) in which gastrointestinal helminth infections did not increase with group size either [11]. Additional studies on the relationship between social group size and dynamics, spatial spread and parasite infestation in proboscis monkeys are desirable to elucidate actual transmission pathways and behavioral strategies of differently sized groups. Further, our data did not support the expectation that the number of juveniles in social groups (= proportion of juveniles) has an effect on parasite risk in those parasites known to predominantly affect juveniles (here: *Strongyloides* and *Ascaris* spp.). It is possible, though, that not all group members were equally represented in collected fecal samples, and that feces of smaller juveniles may not have been spotted and sampled as readily as those of adults due to age-related fecal size differences or lower fecal production rate in juveniles. In other nonhuman primates like olive baboons (*Papio cynocephalus anubis*) and Japanese macaques (*Macaca fuscata*), *Strongyloides* spp. were described to be more prevalent among juveniles [57, 58]. Furthermore, group type was no significant predictor variable of parasite risk in the present study. This result aligns with missing effects by the proportion of juvenile group members on parasite risk, as those are only found in harem groups. Overall, our results do support the increasing number of studies [10, 59, 60] disproving a generalized effect of increased susceptibility of male hosts (or bachelor groups) to parasite infection due to immunosuppressive effects of the sex steroid testosterone [61].

Moreover, we investigated whether seasonal climatic changes shape parameters of parasite risk at both study sites. This study revealed temporal prevalence dynamics only for excretion of *Strongyloides* eggs. A specific nature of this genus is a generational change, which comprises an external non-parasitic and an internal parasitic cycle. From eggs shed in the environment, both infective larvae as well as free-living, fertile adult threadworms can develop. The external life cycle benefits from increasing humidity by a higher cumulative survival of adult generations and larvae in the environment which may then result in higher (re-)infection rates. *Strongyloides* prevalence was shown to increase with enhanced amounts of precipitation in different wildlife species like eastern chimpanzees (*Pan troglodytes schweinfurthii)* [62] or springbok populations (*Antidorcas marsupialis*) [63]. Additionally, higher temperatures rather favor generation of adult threadworms in the environment, multiplying the occurrence of infective larvae. During sampling months, monthly precipitation increased and monthly mean temperature rose towards the end of the year (Fig 2). In accordance, the prevalence of *Strongyloides* spp. significantly increased towards the year’s end. Ongoing studies comprising complete annual seasons at different sampling sites would be desirable to link different parameters of parasite risk to climatic patterns. Summing up, our findings revealed that site of origin outweighed the effects of ecological parameters on parasite risk in proboscis monkeys. Ecological effects tested in the present study (group size, the proportion of juvenile group members per group, group type and seasonal climatic changes) did not explain differences in parasite risk among sampling sites.

No clinical symptoms (such as diarrhea or poor body condition) could be observed in proboscis monkeys inhabiting the LBPMS. However, presented results revealed that an anthropogenically managed area can influence the parasite risk in proboscis monkeys with potentially pathogenic species. Although infestation with *Trichuris* spp. and *Oesophagostomum* spp. usually remains subclinical, fatal whipworm infection with heavy worm burdens, secondary complications like bacterial infection, and stress have proven clinically important resulting in severe enteritis, anorexia or death of the host [45, 64]. Likewise, *Trichuris* spp. infection was described to decrease the population size of a wild nonhuman primate species [5]. Moreover, strongyloidiasis poses a serious health problem in nonhuman primate colonies [65] and was detected as a primary cause of death in rehabilitant orangutans (*Pongo pygmaeus*) [66]. Common symptoms are debilitation, reduced growth rates, dyspnea caused by migrating larvae, and death [45]. Therefore, appropriate strategies and prophylactic measures for proboscis monkeys and other anthropogenically managed wildlife populations infected with detected parasite species are required.

Important management tools to face helminth infections comprise a consistent, noninvasive endoparasite monitoring to identify pathogenic parasites together with a targeted antiparasitic treatment which can reduce parasite infestation and may support breeding success [67]. For different nonhuman primate hosts, effective anthelmintic regimes against detected species are available [45] and strongly suggested to improve welfare. If animals are fed on a daily basis like proboscis monkeys at the LBPMS, regular deworming would be feasible and advisable. However, successful eradication of *Strongyloides* and other soil-transmitted species like *Trichuris* and strongyles requires further strict sanitary practices. To interrupt the external development and survival of these soil-transmitted helminthes in enclosures, surfaces should be kept dry, feces needs to be removed daily from the feeding platforms and surroundings, and contamination of food and water needs to be impeded [45]. Likewise, periodic replacement of soil or rotation of platform use is recommended to prevent from recurrent reinfections [7]. Additionally, appropriate group sizes and overall host densities should be considered to limit stress that is known to increase susceptibility to parasite infections. Reference values for appropriate group sizes of proboscis monkeys and other colobines in captivity are available in specialist guidelines like the expert report on the minimum requirements for the keeping of mammals published by the German Federal Ministry of Food and Agriculture (http://www.bmel.de/EN/Animals/AnimalWelfare.html, retrieved 3^rd^ February 2017).

## Conclusions

In conclusion, our study supported the hypothesis that parasite risk is elevated in anthropogenically modified habitats [68]. The site of origin outweighed the effects of ecological parameters on parasite risk in the proboscis monkey. Especially through increased host density in public sanctuaries and on feeding platforms, endangered proboscis monkeys are at risk of parasitic infections, particularly during the rainy season. Whereas climatic and temporal dynamics are evident in both study sites, group structure and composition had no significant effect for transmission dynamics and disease risk with intestinal parasites. Thus, management strategies for this large endangered primate should focus on regular parasite assessment, consistent deworming procedures and sanitary practices. All in all, this study has provided strong empirical evidence that noninvasive fecal collection represents an important tool for conservation medicine to rapidly assess the intestinal parasite risk of endangered wildlife in tropical environments.

## Supporting information

S1 Table Best models (GLMM) for prevalence of nematode infections and influencing variables among helminth groups found in proboscis monkeys, calculated with binomial presence/absence data. If sampling site had a significant effect, two separate models–one for each sampling site—were calculated per helminth group.
(DOCX)

S2 Table Best models (GLMM) for parasite species richness (PSR) of nematode infections and influencing variables among helminth groups found in proboscis monkeys, calculated under the assumption of a Poisson distribution. If sampling site had a significant effect, two separate models—one for each sampling site—were calculated.
(DOCX)

S3 Table Best linear mixed effect models for log(1+x)-transformed numbers of eggs per gram feces (epg) of nematode infections and influencing variables among helminth groups found in proboscis monkeys. If sampling site had a significant effect, two separate models for sampling sites were calculated per helminth group.
(DOCX)

S1 Appendix Dataset. (XLSX)
