# Supplementary material for: Fecal parasite risk in the endangered proboscis monkey is higher in an anthropogenically managed forest environment compared to a riparian rain forest in Sabah, Borneo
Source: PLoS One. 2018 Apr 9;13(4):e0195584. doi: 10.1371/journal.pone.0195584 (PMC5891069; doi:10.1371/journal.pone.0195584)
Supplement: S1 Table — If sampling site had a significant effect, two separate models–one for each sampling site—were calculated per helminth group. (DOCX) [file pone.0195584.s001.docx]

**S1 Table**

| Parasite tested | Fixed effects | Estimate | Std. Error | Z value | Pr (>\|z\| ) | Effect on prevalence |
| --- | --- | --- | --- | --- | --- | --- |
| Trichurids | **Sampling site LBPMS** | **-1.1214** | **0.5046** | **-2.222** | **0.026*** | **LKWS<LBPMS** |
| LKWS | (Intercept) | -2.8020 | 0.8103 | -3.458 | <0.001** |  |
|  | **Sample weight** | **1.0931** | **0.2476** | **4.414** | **<0.0001***** | **Negative relationship** |
|  | Group type harem | -0.6315 | 0.4384 | -1.440 | 0.150 |  |
|  | Group size | 0.0074 | 0.0337 | 0.219 | 0.827 |  |
|  | No. juveniles <0.3 | 0.5247 | 0.4192 | 1.252 | 0.211 |  |
|  | June | -0.0450 | 0.4816 | -0.093 | 0.926 |  |
|  | July | -0.1791 | 0.5048 | -0.355 | 0.723 |  |
|  | September | -0.4056 | 0.7860 | -0.516 | 0.606 |  |
| LBPMS | (Intercept) | -396.3030 | 81659.4760 | -0.005 | 0.996 |  |
|  | **Sample weight** | **2.6830** | **1.2670** | **2.118** | **0.034*** | **Negative relationship** |
|  | Group type harem | -311.7260 | 73204.8510 | -0.004 | 0.997 |  |
|  | Group size | 19.4930 | 4264.3870 | 0.005 | 0.996 |  |
|  | No. juveniles <0.3 | 292.3170 | 63965.7990 | 0.005 | 0.996 |  |
|  | September | 1.9800 | 1.0340 | 1.915 | 0.056. |  |
| Trichuris sp. T1 | Sampling site LBPMS | -0.4150 | 0.6451 | -0.643 | 0.520 |  |
|  | (Intercept) | 2.5389 | 1.3441 | 1.889 | 0.059. |  |
|  | **Sample weight** | **1.4319** | **0.2761** | **5.185** | **<0.0001***** | **Negative relationship** |
|  | Group type harem | -1.0935 | 0.9258 | -1.181 | 0.238 |  |
|  | Group size | -0.0634 | 0.0382 | -1.660 | 0.097. |  |
|  | No. juveniles <0.3 | -0.0634 | 0.6933 | -0.091 | 0.927 |  |
|  | June | -0.3840 | 0.9392 | -0.409 | 0.683 |  |
|  | July | -0.4850 | 0.9893 | -0.490 | 0.624 |  |
|  | September | 0.2150 | 1.1659 | 0.184 | 0.854 |  |
|  | October | 0.6472 | 1.2919 | 0.501 | 0.616 |  |
| Trichuris sp. T2 | Sampling site LBPMS | -0.2661 | 0.4595 | -0.579 | 0.563 |  |
|  | (Intercept) | -2.9088 | 0.7241 | -4.017 | <0.0001*** |  |
|  | **Sample weight** | **0.9765** | **0.2163** | **4.514** | **<0.0001***** | **Negative relationship** |
|  | Group type harem | -0.4029 | 0.4177 | -0.965 | 0.335 |  |
|  | Group size | 0.0296 | 0.0229 | 1.295 | 0.195 |  |
|  | No. juveniles <0.3 | 0.5880 | 0.3690 | 1.594 | 0.111 |  |
|  | June | -0.1935 | 0.4506 | -0.430 | 0.668 |  |
|  | July | -0.0145 | 0.4457 | -0.032 | 0.974 |  |
|  | September | 0.4527 | 0.5797 | 0.781 | 0.435 |  |
|  | October | -0.4811 | 0.7378 | -0.652 | 0.514 |  |
| Trichuris sp. T3 | **Sampling site LBPMS** | **-2.2722** | **0.4447** | **-5.110** | **<0.0001***** | **LKWS<LBPMS** |
| LKWS | (Intercept) | 1.4349 | 0.9812 | 1.462 | 0.144 |  |
|  | **Sample weight** | **0.6861** | **0.1969** | **3.485** | **0.001**** | **Negative relationship** |
|  | Group type harem | -0.0322 | 0.5894 | -0.055 | 0.957 |  |
|  | Group size | 0.0533 | 0.0411 | -1.296 | 0.195 |  |
|  | No. juveniles <0.3 | -0.7179 | 0.5235 | -1.371 | 0.170 |  |
|  | June | -0.3690 | 0.6585 | -0.560 | 0.575 |  |
|  | July | -0.9594 | 0.7128 | -1.346 | 0.178 |  |
|  | September | -0.8163 | 0.8778 | -0.930 | 0.352 |  |
| LBPMS | (Intercept) | 276.7704 | 1451.2209 | 0.191 | 0.849 |  |
|  | **Sample weight** | **3.3373** | **1.2683** | **2.631** | **0.009*** | **Negative relationship** |
|  | Group type harem | 287.1204 | 1561.6835 | 0.184 | 0.854 |  |
|  | Group size | -15.8000 | 18.8566 | -0.838 | 0.402 |  |
|  | No. juveniles <0.3 | -238.8774 | 282.8543 | -0.844 | 0.398 |  |
|  | September | 0.2228 | 0.7614 | 0.293 | 0.770 |  |
| Anatrichosoma spp. | Sampling site LBPMS | -0.6687 | 0.7920 | -0.844 | 0.398 |  |
|  | (Intercept) | 22.4274 | 117.0783 | 0.192 | 0.848 |  |
|  | Group type harem | 0.3075 | 1.2259 | 0.251 | 0.802 |  |
|  | Group size | -0.0022 | 0.0718 | -0.030 | 0.976 |  |
|  | No. juveniles <0.3 | -1.1441 | 0.9413 | -1.216 | 0.224 |  |
|  | June | -18.0139 | 117.0796 | -0.154 | 0.878 |  |
|  | July | 18.4712 | 117.0784 | -0.158 | 0.875 |  |
|  | September | -18.9404 | 117.0873 | -0.162 | 0.871 |  |
|  | October | -19.1460 | 117.0942 | -0.164 | 0.870 |  |
| Strongylids | Sampling site LBPMS | -0.0633 | 0.4358 | -0.145 | 0.885 |  |
|  | (Intercept) | -0.6846 | 0.8047 | -0.851 | 0.395 |  |
|  | Group type harem | -0.1157 | 0.4940 | -0.234 | 0.815 |  |
|  | Group size | 0.0307 | 0.0262 | 1.170 | 0.242 |  |
|  | No. juveniles <0.3 | 0.0924 | 0.4939 | 0.187 | 0.852 |  |
|  | June | 0.3077 | 0.6454 | 0.477 | 0.634 |  |
|  | July | 0.0004 | 0.6640 | 0.001 | 0.999 |  |
|  | September | -0.5050 | 0.7401 | -0.682 | 0.495 |  |
|  | October | -0.3462 | 0.8494 | -0.408 | 0.684 |  |
| *Trichostrongylus* spp. | Sampling site LBPMS | -0.1617 | 0.4309 | -0.375 | 0.708 |  |
|  | (Intercept) | 0.2826 | 0.8300 | 0.340 | 0.734 |  |
|  | Group type harem | -0.1764 | 0.5208 | -0.339 | 0.735 |  |
|  | Group size | 0.0285 | 0.0265 | 1.075 | 0.283 |  |
|  | No. juveniles <0.3 | -0.2368 | 0.4967 | -0.477 | 0.633 |  |
|  | June | -0.0557 | 0.6460 | -0.086 | 0.931 |  |
|  | July | -0.3051 | 0.6657 | -0.458 | 0.647 |  |
|  | September | -0.8510 | 0.7562 | -1.125 | 0.260 |  |
|  | October | -0.4303 | 0.8731 | -0.493 | 0.622 |  |
| *Oesophagostomum*/*Ternidens* spp. | **Sampling site LBPMS** | **-1.1644** | **0.4456** | **-2.613** | **0.009*** | **LKWS<LBPMS** |
| LKWS | (Intercept) | 0.3796 | 0.8361 | 0.454 | 0.650 |  |
|  | Group type harem | 0.3561 | 0.4735 | 0.752 | 0.452 |  |
|  | Group size | 0.0247 | 0.0353 | 0.699 | 0.484 |  |
|  | No. juveniles <0.3 | 0.1013 | 0.4555 | 0.222 | 0.824 |  |
|  | June | 0.3931 | 0.5587 | 0.704 | 0.482 |  |
|  | July | 0.2323 | 0.6035 | 0.385 | 0.700 |  |
|  | September | -0.3125 | 0.7474 | -0.418 | 0.676 |  |
| LBPMS | (Intercept) | -5.2092 | 5.4253 | -0.960 | 0.337 |  |
|  | Group type harem | -6.2396 | 7.0645 | -0.883 | 0.377 |  |
|  | Group size | 0.3466 | 0.3439 | 1.008 | 0.314 |  |
|  | No. juveniles <0.3 | 4.2551 | 5.3642 | 0.793 | 0.428 |  |
|  | September | -0.1681 | 0.6854 | -0.245 | 0.806 |  |
| Unknown strongylid | Sampling site LBPMS | -1.0960 | 0.7689 | -1.425 | 0.154 |  |
|  | (Intercept) | 20.3643 | 6875.1804 | 0.003 | 0.998 |  |
|  | **Sample weight** | **1.0616** | **0.5134** | **2.068** | **0.039*** | **Negative relationship** |
|  | Group type harem | 0.0675 | 1.0036 | 0.067 | 0.946 |  |
|  | Group size | 0.0136 | 0.0486 | 0.280 | 0.779 |  |
|  | No. juveniles <0.3 | -0.4073 | 0.9095 | -0.448 | 0.654 |  |
|  | June | -17.8540 | 6875.1803 | -0.003 | 0.998 |  |
|  | July | -18.2519 | 6875.1803 | -0.003 | 0.998 |  |
|  | September | -19.9213 | 6875.1804 | -0.003 | 0.998 |  |
|  | October | -18.9962 | 6875.1804 | -0.003 | 0.998 |  |
| *Strongyloides* spp. | **Sampling site LBPMS** | **-1.6807** | **0.4477** | **-3.754** | **<0.001**** | **LKWS<LBPMS** |
| LKWS | (Intercept) | 11.0923 | 6.4211 | 1.728 | 0.084. |  |
|  | **Sample weight** | **-0.4810** | **0.1813** | **-2.654** | **0.008*** | **Positive relationship** |
|  | Group type harem ^a^ | -0.8153 | 0.7946 | -1.026 | 0.305 |  |
|  | Group size | -0.8169 | 0.7349 | -1.112 | 0.266 |  |
|  | No. juveniles <0.3 | -4.6613 | 6.1590 | -0.757 | 0.449 |  |
|  | No. juveniles >0.3 | -7.6781 | 6.1929 | -1.240 | 0.215 |  |
|  | **June** | **-2.1421** | **1.0641** | **-2.013** | **0.044*** | **June< September, August<September** ^b^ |
|  | **July** | **-2.6083** | **1.1758** | **-2.218** | **0.027*** |  |
|  | **September** | **-3.6411** | **1.2094** | **-3.011** | **0.003*** |  |
|  | Group size X No. juveniles <0.3 | 0.3430 | 0.7308 | 0.469 | 0.639 |  |
|  | Group size X No. juveniles >0.3 | 0.8549 | 0.7310 | 1.170 | 0.242 |  |
| LBPMS | (Intercept) | -9.079 | 5.238 | -1.733 | 0.083. |  |
|  | **Sample weight** | **1.734** | **0.8000** | **2.180** | **0.029*** | **Positive relationship** |
|  | Group type | -62.2700 | 36.9000 | -1.687 | 0.092. |  |
|  | Group size | 0.2572 | 0.3081 | 0.835 | 0.404 |  |
|  | No. juveniles <0.3 | 29.1800 | 7915000.0 | 0.000 | 1.000 |  |
|  | **September** | **1.2860** | **0.5952** | **2.160** | **0.031*** | **September<October** |
|  | Group size X No. juveniles >0.3 | 1.6410 | 1.0640 | 1.543 | 0.123 |  |
| *Ascaris lumbricoides* | Sampling site LBPMS | 1.7101 | 1.0337 | 1.654 | 0.098. |  |
|  | (Intercept) | 3.3859 | 1.4348 | 2.360 | 0.018* |  |
|  | Group type harem | 0.8442 | 0.6480 | 1.303 | 0.193 |  |
|  | Group size | 0.0559 | 0.0465 | 1.202 | 0.229 |  |
|  | No. juveniles <0.3 | -0.7220 | 0.6140 | -1.176 | 0.240 |  |
|  | June | -2.1774 | 1.2347 | -1.764 | 0.078. |  |
|  | July | -1.6991 | 1.2498 | -1.359 | 0.174 |  |
|  | September | -1.8111 | 1.4442 | -1.254 | 0.210 |  |
|  | October | 14.4080 | 3430.7603 | 0.004 | 0.997 |  |
| *Enterobius* spp.^c^ | (Intercept) | -13.7995 | 3774.7056 | -0.004 | 0.997 |  |
|  | Group type harem | -1.0327 | 1.3165 | -0.784 | 0.433 |  |
|  | Group size | -0.0385 | 0.0621 | -0.620 | 0.535 |  |
|  | No. juveniles <0.3 | 17.8016 | 3774.7053 | 0.005 | 0.996 |  |
|  | June | 17.8533 | 3774.7053 | 0.005 | 0.996 |  |
|  | July | 18.3641 | 3774.7054 | 0.005 | 0.996 |  |
|  | September | 36.6024 | 7931.3141 | 0.005 | 0.996 |  |

Significant associations are marked with asterisks (* p≤0.05; ** p≤0.001; *** p≤0.0001).

LKWS=Lower Kinabatangan Wildlife Sanctuary, LBPMS=Labuk Bay Proboscis Monkey Sanctuary

No. juveniles=Number of juveniles (given as the proportion of juvenile group members) per group

^a^ tested in a model without interaction term group size X No. juveniles

^b^ a post hoc test for all pairwise comparisons between sampling months in the LKWS revealed significant differences in prevalence of *Strongyloides* spp. between June and September (p=0.0231) as well as between August and September (p=0.0117)

^c^ only observed in fecal samples from the LKWS
