# Supplementary material for: Fecal parasite risk in the endangered proboscis monkey is higher in an anthropogenically managed forest environment compared to a riparian rain forest in Sabah, Borneo
Source: PLoS One. 2018 Apr 9;13(4):e0195584. doi: 10.1371/journal.pone.0195584 (PMC5891069; doi:10.1371/journal.pone.0195584)
Supplement: S2 Table — If sampling site had a significant effect, two separate models—one for each sampling site—were calculated. (DOCX) [file pone.0195584.s002.docx]

**S2 Table**

| Dependent variable | Fixed effects | Estimate | Std. Error | Z value | Pr (>\|z\| ) | Impact on PSR |
| --- | --- | --- | --- | --- | --- | --- |
| **PSR** | **Sampling site LBPMS** | **0.3489** | **0.1187** | **2.939** | **0.003*** | **LKWS<**  **LBPMS** |
| LKWS | (Intercept) | -0.2233 | 0.7776 | -0.287 | 0.774 |  |
|  | Group type harem | 0.5238 | 0.7616 | 0.688 | 0.492 |  |
|  | Group size | 0.0553 | 0.0996 | 0.555 | 0.579 |  |
|  | No. juveniles <0.3 | -0.5166 | 0.3014 | -1.714 | 0.087. |  |
|  | **June** | **0.5860** | **0.2941** | **1.993** | **0.046*** | **Trend for August<**  **September ^a^** |
|  | **July** | **0.6800** | **0.3365** | **2.021** | **0.043*** |  |
|  | **September** | **0.7966** | **0.3408** | **2.337** | **0.019*** |  |
|  | Group size X No. juveniles <0.3 | 0.0283 | 0.1136 | 0.249 | 0.804 |  |
|  | Group size X No. juveniles >0.3 | -0.0635 | 0.1016 | -0.624 | 0.532 |  |
| LBPMS | (Intercept) | 9.3243 | 10.3787 | 0.898 | 0.369 |  |
|  | Group type harem | 6.7353 | 9.2731 | 0.726 | 0.468 |  |
|  | Group size | -0.4192 | 0.5464 | -0.767 | 0.443 |  |
|  | No. juveniles <0.3 | -5.9122 | 8.1883 | -0.722 | 0.470 |  |
|  | September | -0.1903 | 0.1960 | -0.971 | 0.331 |  |

Significant associations are marked with asterisks (* p≤0.05; ** p≤0.001; *** p≤0.0001).

LKWS=Lower Kinabatangan Wildlife Sanctuary, LBPMS=Labuk Bay Proboscis Monkey Sanctuary

No. juveniles=Number of juveniles (given as the proportion of juvenile group members) per group

^a^ a post hoc test for all pairwise comparisons between sampling months only revealed a trend for PSR in the LKWS to be higher in September (mean PSR 2.6 ± 1.2) compared to August (mean PSR 2.2 ± 1.3) (p=0.0783)
