# Supplementary material for: Fecal parasite risk in the endangered proboscis monkey is higher in an anthropogenically managed forest environment compared to a riparian rain forest in Sabah, Borneo
Source: PLoS One. 2018 Apr 9;13(4):e0195584. doi: 10.1371/journal.pone.0195584 (PMC5891069; doi:10.1371/journal.pone.0195584)
Supplement: S3 Table — If sampling site had a significant effect, two separate models for sampling sites were calculated per helminth group. (DOCX) [file pone.0195584.s003.docx]

**S3 Table**

| Parasite tested | Fixed effects | Estimate | Std. Error | DF | T value | Pr (>\|t\|) | Effect on epg |
| --- | --- | --- | --- | --- | --- | --- | --- |
| Trichurids | Sampling site LBPMS | 0.7039 | 0.3676 | 40.1100 | 1.915 | 0.063. |  |
|  | (Intercept) | 1.9855 | 0.8797 | 15.7730 | 2.257 | 0.039* |  |
|  | Group type harem | -0.2198 | 0.5077 | 18.9810 | -0.433 | 0.670 |  |
|  | Group size | -0.0083 | 0.0286 | 15.3720 | -0.289 | 0.776 |  |
|  | No. juveniles <0.3 | -0.0290 | 0.5605 | 15.0610 | -0.052 | 0.959 |  |
|  | June | 0.7240 | 0.7502 | 13.4540 | 0.965 | 0.352 |  |
|  | July | 0.3476 | 0.7678 | 13.4830 | 0.453 | 0.658 |  |
|  | September | 1.0688 | 0.8113 | 15.6300 | 1.317 | 0.207 |  |
|  | October | 1.4032 | 0.8515 | 19.1740 | 1.648 | 0.116 |  |
| Trichuris sp. T1 | Sampling site LBPMS | -0.0512 | 0.0826 | 47.1700 | -0.620 | 0.538 |  |
|  | (Intercept) | -0.0207 | 0.1808 | 17.2140 | -0.114 | 0.910 |  |
|  | Group type harem | 0.0585 | 0.1117 | 22.1560 | 0.524 | 0.606 |  |
|  | Group size | 0.0017 | 0.0061 | 17.3050 | 0.273 | 0.788 |  |
|  | No. juveniles <0.3 | -0.0082 | 0.1072 | 14.3170 | -0.077 | 0.940 |  |
|  | June | 0.1381 | 0.1401 | 13.2910 | 0.986 | 0.342 |  |
|  | July | 0.0513 | 0.1451 | 13.3850 | 0.354 | 0.729 |  |
|  | September | 0.0782 | 0.1639 | 18.0020 | 0.477 | 0.639 |  |
|  | October | 0.0057 | 0.1820 | 25.9760 | 0.031 | 0.975 |  |
| Trichuris sp. T2 | Sampling site LBPMS | 0.3605 | 0.4220 | 37.3000 | 0.854 | 0.398 |  |
|  | (Intercept) | 2.0505 | 1.0044 | 13.3280 | 2.042 | 0.062. |  |
|  | Group type harem | 0.3114 | 0.5915 | 16.5150 | 0.526 | 0.606 |  |
|  | Group size | -0.0214 | 0.0339 | 13.1730 | -0.630 | 0.539 |  |
|  | No. juveniles <0.3 | -0.5399 | 0.6093 | 12.8030 | -0.886 | 0.392 |  |
|  | June | 0.2809 | 0.8155 | 11.2470 | 0.344 | 0.737 |  |
|  | July | -0.0562 | 0.8434 | 11.2760 | 0.067 | 0.948 |  |
|  | September | -0.0570 | 0.9042 | 13.7350 | -0.063 | 0.951 |  |
|  | October | 0.7745 | 0.9518 | 16.8870 | 0.814 | 0.427 |  |
| Trichuris sp. T3 | **Sampling site LBPMS** | **1.1920** | **0.2118** | **20.0170** | **5.627** | **<0.0001***** | **LKWS<LBPMS** |
| LKWS | (Intercept) | -0.0922 | 0.4003 | 9.8380 | -0.230 | 0.823 |  |
|  | Group type harem | 0.0026 | 0.2329 | 11.1380 | 0.011 | 0.991 |  |
|  | Group size | -0.0013 | 0.0167 | 9.1280 | -0.078 | 0.939 |  |
|  | No. juveniles <0.3 | 0.2962 | 0.2405 | 8.3750 | 1.232 | 0.251 |  |
|  | June | 0.4145 | 0.2966 | 8.3710 | 1.398 | 0.198 |  |
|  | July | 0.3309 | 0.3127 | 8.6620 | 1.058 | 0.318 |  |
|  | September | 0.5830 | 0.3703 | 9.8050 | 1.574 | 0.147 |  |
| LBPMS | (Intercept) | -17.3647 | 27.6108 | 0.0000 | -0.629 | 0.991 |  |
|  | Group type harem | -21.0229 | 24.4023 | 0.0000 | -0.862 | 0.989 |  |
|  | Group size | 1.0968 | 1.4529 | 0.0000 | 0.755 | 0.990 |  |
|  | No. juveniles <0.3 | 17.5620 | 21.5090 | 0.0000 | 0.816 | 0.989 |  |
|  | September | 0.0170 | 0.3305 | 44.0000 | 0.051 | 0.959 |  |
| Anatrichosoma spp. | Sampling site LBPMS | 0.0006 | 0.0011 | 684.0000 | 0.546 | 0.585 |  |
|  | (Intercept) | -0.0771 | 0.1267 | 6.4450 | -0.609 | 0.563 |  |
|  | Group type harem | 0.0199 | 0.0714 | 6.7280 | 0.278 | 0.789 |  |
|  | Group size | 0.0054 | 0.0043 | 6.6860 | 1.258 | 0.251 |  |
|  | No. juveniles <0.3 | -0.0069 | 0.0765 | 6.6020 | -0.090 | 0.931 |  |
|  | June | 0.0025 | 0.1072 | 6.0990 | 0.023 | 0.982 |  |
|  | July | 0.0416 | 0.1109 | 6.0940 | 0.375 | 0.720 |  |
|  | September | 0.0377 | 0.1131 | 6.5080 | 0.333 | 0.749 |  |
|  | October | 0.0455 | 0.1144 | 6.8080 | 0.397 | 0.703 |  |
| Strongylids | Sampling site LBPMS | 0.3222 | 0.1928 | 31.8200 | 1.671 | 0.105 |  |
|  | (Intercept) | 0.5352 | 0.4212 | 14.6670 | 1.271 | 0.224 |  |
|  | Group type harem | 0.1033 | 0.2416 | 17.2640 | 0.428 | 0.674 |  |
|  | Group size | -0.0129 | 0.0137 | 14.1560 | -0.940 | 0.363 |  |
|  | No. juveniles <0.3 | 0.0359 | 0.2687 | 14.2950 | 0.133 | 0.896 |  |
|  | June | 0.1707 | 0.3612 | 12.7050 | 0.473 | 0.645 |  |
|  | July | 0.1391 | 0.3697 | 12.7120 | 0.376 | 0.713 |  |
|  | September | 0.5454 | 0.3886 | 14.6060 | 1.404 | 0.181 |  |
|  | October | 0.5557 | 0.4051 | 17.4480 | 1.372 | 0.187 |  |
| *Trichostrongylus* spp. | Sampling site LBPMS | 0.0943 | 0.1580 | 34.8700 | 0.597 | 0.554 |  |
|  | (Intercept) | 0.3140 | 0.3446 | 14.2620 | 0.911 | 0.377 |  |
|  | Group type harem | 0.1642 | 0.2021 | 17.9350 | 0.812 | 0.427 |  |
|  | Group size | -0.0126 | 0.0113 | 14.0980 | -1.115 | 0.283 |  |
|  | No. juveniles <0.3 | 0.0741 | 0.2189 | 13.3360 | 0.339 | 0.740 |  |
|  | June | 0.1623 | 0.2918 | 11.8610 | 0.556 | 0.589 |  |
|  | July | 0.1261 | 0.2990 | 11.9390 | 0.422 | 0.681 |  |
|  | September | 0.4023 | 0.3180 | 14.2150 | 1.265 | 0.226 |  |
|  | October | 0.3575 | 0.3348 | 17.5970 | 1.068 | 0.300 |  |
| ***Oesophagostomum*/*Ternidens* spp.** | **Sampling site LBPMS** | **0.5151** | **0.1227** | **28.4290** | **4.199** | **<0.001**** | **LKWS<LBPMS** |
| LKWS | (Intercept) | 0.4276 | 0.2596 | 9.1530 | 1.647 | 0.133 |  |
|  | Group type harem | -0.0279 | 0.1503 | 10.1690 | -0.185 | 0.857 |  |
|  | Group size | -0.0112 | 0.0108 | 8.4940 | -1.040 | 0.327 |  |
|  | No. juveniles <0.3 | -0.0912 | 0.1533 | 7.4570 | -0.595 | 0.570 |  |
|  | June | -0.0303 | 0.1889 | 7.4270 | -0.160 | 0.877 |  |
|  | July | -0.0808 | 0.2002 | 7.8020 | -0.404 | 0.697 |  |
|  | September | -0.0239 | 0.2372 | 8.9090 | -0.101 | 0.922 |  |
| LBPMS | (Intercept) | 1.6984 | 2.6007 | 1.3300 | 0.653 | 0.608 |  |
|  | Group type harem | 1.5688 | 3.2381 | 1.4200 | 0.484 | 0.692 |  |
|  | Group size | -0.0750 | 0.1609 | 1.3800 | -0.466 | 0.704 |  |
|  | No. juveniles <0.3 | -1.0000 | 2.4980 | 1.4100 | -0.400 | 0.741 |  |
|  | September | 0.0013 | 0.1610 | 59.3500 | 0.009 | 0.993 |  |
| Unknown strongylid | Sampling site LBPMS | 0.0039 | 0.0217 | 25.1520 | 0.180 | 0.859 |  |
|  | (Intercept) | 0.0175 | 0.0417 | 14.6400 | 0.420 | 0.681 |  |
|  | Group type harem | 0.0158 | 0.0266 | 16.6380 | 0.595 | 0.560 |  |
|  | Group size | -0.0024 | 0.0014 | 18.1830 | -1.687 | 0.107 |  |
|  | No. juveniles <0.3 | -0.0052 | 0.0244 | 9.9420 | -0.215 | 0.834 |  |
|  | June | 0.0297 | 0.0318 | 10.3850 | 0.935 | 0.371 |  |
|  | July | 0.0083 | 0.0328 | 10.5710 | 0.252 | 0.806 |  |
|  | September | 0.0814 | 0.0387 | 16.3340 | 2.104 | 0.051. |  |
|  | October | 0.0629 | 0.0456 | 24.6530 | 1.380 | 0.180 |  |
| *Strongyloides* spp. | **Sampling site LBPMS** | **1.0255** | **0.1696** | **22.1110** | **6.047** | **<0.0001***** | **LKWS<LBPMS** |
| LKWS | (Intercept) | -0.4849 | 0.6397 | 14.6950 | -0.758 | 0.460 |  |
|  | Group type harem | 0.1759 | 0.6101 | 17.1860 | 0.288 | 0.777 |  |
|  | Group size | 0.0035 | 0.0822 | 14.4770 | 0.043 | 0.967 |  |
|  | No. juveniles <0.3 | -0.7622 | 0.3439 | 5.7630 | -2.217 | 0.070. |  |
|  | June | 0.4709 | 0.3261 | 6.0680 | 1.444 | 0.198 |  |
|  | July | 0.6812 | 0.3708 | 6.0760 | 1.837 | 0.115 |  |
|  | September | 0.8771 | 0.3662 | 6.4580 | 2.395 | 0.051. |  |
|  | Group size X No. juveniles <0.3 | 0.1364 | 0.1021 | 10.2780 | 1.336 | 0.210 |  |
|  | Group size X No. juveniles >0.3 | 0.0004 | 0.0849 | 13.7740 | 0.004 | 0.997 |  |
| LBPMS | (Intercept) | 2.1716 | 2.2476 | 0.0000 | 0.966 | 0.998 |  |
|  | Group type harem | 31.4386 | 20.9551 | 0.0000 | 1.500 | 1.000 |  |
|  | Group size | -0.0518 | 0.1419 | 0.0000 | -0.365 | 0.999 |  |
|  | No. juveniles <0.3 | -31.3906 | 21.0537 | 0.0000 | -1.491 | 1.000 |  |
|  | September | -0.4733 | 0.2462 | 62.0000 | -1.922 | 0.059. |  |
|  | Group size X No. juveniles >0.3 | -0.8545 | 0.6010 | 0.0000 | -1.422 | 1.000 |  |
| *Ascaris lumbricoides* | Sampling site LBPMS | -0.0629 | 0.0411 | 47.4900 | -1.530 | 0.133 |  |
|  | (Intercept) | 0.0525 | 0.07672 | 13.2540 | 0.684 | 0.506 |  |
|  | Group type harem | -0.0159 | 0.04638 | 17.2710 | -0.344 | 0.735 |  |
|  | Group size | -0.0039 | 0.0025 | 14.1620 | -1.547 | 0.144 |  |
|  | No. juveniles <0.3 | 0.0189 | 0.0476 | 10.6570 | 0.397 | 0.699 |  |
|  | June | 0.0913 | 0.0625 | 10.1760 | 1.461 | 0.174 |  |
|  | July | 0.0348 | 0.0641 | 10.2980 | 0.543 | 0.599 |  |
|  | September | 0.0670 | 0.0704 | 12.8660 | 0.952 | 0.359 |  |
|  | October | 0.0486 | 0.0783 | 19.1470 | 0.621 | 0.542 |  |
| *Enterobius* spp.^a^ | (Intercept) | 0.0848 | 0.0847 | 19.9850 | 1.002 | 0.328 |  |
|  | Group type harem | 0.0483 | 0.0519 | 25.5030 | 0.930 | 0.361 |  |
|  | Group size | 0.0003 | 0.0028 | 22.0540 | 0.115 | 0.909 |  |
|  | No. juveniles <0.3 | 0.0701 | 0.0518 | 15.2220 | -1.353 | 0.196 |  |
|  | June | -0.0486 | 0.0679 | 14.9990 | -0.716 | 0.485 |  |
|  | July | -0.0725 | 0.0696 | 15.1880 | -1.041 | 0.314 |  |
|  | September | -0.1330 | 0.0776 | 19.5990 | -1.713 | 0.103 |  |

Significant associations are marked with asterisks (* p≤0.05; ** p≤0.001; *** p≤0.0001).

LKWS=Lower Kinabatangan Wildlife Sanctuary, LBPMS=Labuk Bay Proboscis Monkey Sanctuary

DF=degrees of freedom

No. juveniles=Number of juveniles (given as the proportion of juvenile group members) per group

^a^ only observed in fecal samples from the LKWS
